# Anti-NMDA Receptor Encephalitis or Psychiatric disorder?

**DOI:** 10.1192/j.eurpsy.2022.2289

**Published:** 2022-09-01

**Authors:** B. Freitas, M.D.C. Vasconcelos, F. Ramalheira, D. Terêncio, C. Moreira

**Affiliations:** Centro Hospitalar Psiquiátrico de Lisboa, Psiquiatria, Lisboa, Portugal

**Keywords:** NMDA Receptor Encephalitis, psychiatric symptoms

## Abstract

**Introduction:**

Anti-N-methyl-D-aspartate (NMDA) receptor encephalitis commonly begins with a prodromal phase characterized by flu-like symptoms, subsequently the patients experience a rapid deterioration with psychiatric symptoms that may include anxiety, irritability, insomnia, paranoia, aggression, auditory or visual hallucinations, sexual disinhibition, mania, cognitive disorder, and psychosis; seizures; motor and autonomic dysfunction. The triggers of the disorder comprise viral infections, tumors, and other unknown factors. Taking in count the prominence of psychiatric symptoms, it is relevant to rise the question whether patients with anti-NMDA receptor encephalitis are being misdiagnosed with psychiatric disorders.

**Objectives:**

Non-systematic literature review of the relationship between anti-NMDA receptor encephalitis and psychiatric disorders.

**Methods:**

From the review performed, 2 studies stand out: In one study, 459 serum samples for NMDA receptor antibodies were evaluated. The analysis compared samples from patients diagnosed with schizophrenia, major depressive disorder, and borderline personality disorder with nonpsychiatric controls. In another study, serum was obtained prospectively from a cohort (n = 46) of patients at first presentation of psychosis and NMDA receptor antibodies were measured.

**Results:**

In the first study, the authors found that 9.9%, 2.8%, and 0% of patients with schizophrenia, major depressive disorder, and borderline personality disorder, respectively, were antibody positive. In the second study, it was found that 7% of the patients were serum NMDA receptor antibody positive.

**Conclusions:**

It is unclear yet if patients with primary psychotic disorders have higher rates of pathogenic NMDA receptor antibodies. More evidence is needed to study this relationship.

**Disclosure:**

No significant relationships.

